# Rising Trends in Metabolically Healthy Obesity in Cancer Patients and Its Impact on Cardiovascular Events: Insights from a Contemporary Nationwide Analysis in the USA (2016–2020)

**DOI:** 10.3390/jcm13102820

**Published:** 2024-05-10

**Authors:** Vamsikalyan Borra, Akhil Jain, Nithya Borra, Lakshmi Prasanna Vaishnavi Kattamuri, Sidhartha Gautam Senapati, Naga Vamsi Krishna Machineni, Sindhuja Kukkala, Karthikeya Ramasahayam, Kesar Prajapati, Ankit Vyas, Rupak Desai

**Affiliations:** 1 Department of Internal Medicine, The University of Texas, Rio Grande Valley, Edinburg, TX 78539, USA; borravamsikalyan@gmail.com; 2 Department of Leukemia, The University of Texas MD Anderson Cancer Center, Houston, TX 77079, USA; 3 Department of Internal Medicine, Sri Venkateswara Medical College, Tirupati 517507, India; 4 Department of Internal Medicine, Health Sciences Center, Texas Tech University, El Paso, TX 79409, USA; 5 Department of Pulmonary and Critical Care Medicine, The Johns Hopkins University, Baltimore, MD 21218, USA; 6 Department of Internal Medicine, St. Luke’s Hospital, St. Louis, MO 63122, USA; 7 Konaseema Institute of Medical Sciences and Research Foundation, Amalapuram 533201, India; 8 Department of Internal Medicine, Metropolitan Hospital Center, NYC Health+ Hospitals, New York, NY 11373, USA; 9 Department of Vascular Medicine, Oschner Clinic Foundation, New Orleans, LA 70124, USA; drankitgvyas@gmail.com; 10 Independent Outcomes Researcher, Atlanta, GA 30033, USA; drrupakdesai@gmail.com

**Keywords:** metabolically healthy obesity, cancer, malignancy, major adverse cardiovascular and cerebrovascular events, acute myocardial infarction, cardiac arrest, acute ischemic stroke, mortality

## Abstract

**Background:** Obesity or overweight raises the risk of developing 13 types of cancer, representing 40% of all cancers diagnosed in the United States annually. Given the ongoing debate surrounding the impact of metabolically healthy obesity (MHO) on cardiovascular outcomes, it is crucial to comprehend the incidence of Major Adverse Cardiovascular and Cerebrovascular Events (MACCEs) and the influence of MHO on these outcomes in cancer patients. **Methods:** Data of hospitalized cancer patients with and without obesity were analyzed from the National Inpatient Sample 2016–2020. Metabolically healthy patients were identified by excluding diabetes, hypertension, and hyperlipidemia using Elixhauser comorbidity software, v.2022.1. After that, we performed a multivariable regression analysis for in-hospital MACCEs and other individual outcomes. **Results:** We identified 3,111,824 cancer-related hospitalizations between 2016 and 2020. The MHO cohort had 199,580 patients (6.4%), whereas the MHnO (metabolically healthy non-obese) cohort had 2,912,244 patients (93.6%). The MHO cohort had a higher proportion of females, Blacks, and Hispanics. Outcomes including in-hospital MACCEs (7.9% vs. 9.5%; *p* < 0.001), all-cause mortality (6.1% vs. 7.5%; *p* < 0.001), and acute myocardial infarction (AMI) (1.5% vs. 1.6%; *p* < 0.001) were lower in the MHO cohort compared to the MHnO cohort. Upon adjusting for the baseline characteristics, the MHO group had lower odds of in-hospital MACCEs [adjusted odds ratio (AOR) = 0.93, 95% CI (0.90–0.97), *p* < 0.001], all-cause mortality [AOR = 0.91, 95% CI (0.87–0.94); *p* < 0.001], and acute ischemic stroke (AIS) [AOR = 0.76, 95% CI (0.69–0.84); *p* < 0.001], whereas there were higher odds of acute myocardial infarction (AMI) [AOR = 1.08, 95% CI (1.01–1.16); *p* < 0.001] and cardiac arrest (CA) [AOR = 1.26, 95% CI (1.01–1.57); *p* = 0.045] in the MHO cohort compared to the MHnO cohort. **Conclusions:** Hospitalized cancer patients with MHO exhibited a lower prevalence of in-hospital MACCEs than those with MHnO. Additional prospective studies and randomized clinical trials are imperative to validate these findings, particularly in stratifying MHO across various cancer types and their corresponding risks of in-hospital MACCEs.

## 1. Introduction

A BMI score of 18.5 to 24.9 is considered normal. Overweight is defined as a BMI of 25 to 29.9, and obese as a BMI greater than or equal to 30 [1]. Obesity increases the risk of developing 13 types of cancers (adenocarcinoma of the esophagus, breast, colorectal, and uterus, etc.) [2]. These cancers constitute 40% of all cancers diagnosed in the United States annually. From 2005 to 2014, the rate of new cancers associated with overweight and obesity increased by 7% [2]. Researchers have linked altered fatty acid secretion and metabolism, extracellular matrix remodeling, the secretion of anabolic and sex hormones, immune dysregulation, chronic inflammation, and changes in the gut microbiome to carcinogenesis in obese patients [3,4]. While cancer continues to be a leading cause of morbidity and mortality globally, obesity has emerged as a pervasive and escalating health concern, affecting millions worldwide [5]. Recent surveys have found that approximately 42% of adults and roughly 20% of children and adolescents have obesity [2]. Obesity also contributes to an elevated cardiovascular risk by releasing inflammatory mediators that increase oxidative stress and cause endothelial dysfunction [6]. Obesity-associated inflammation and insulin resistance promote both cardiovascular diseases (CVDs) and cancer [7,8]. Furthermore, proinflammatory states can lead to the development of both CVD and cancer [7,8]. This traditional perception was defied by the emergence of metabolically healthy obesity (MHO), a separate subgroup of obesity that showed decreased cardiovascular risk. 

The concept of metabolically healthy obesity (MHO) originated in the 1950s; however, despite a long-standing recognition of MHO, a unified definition has yet to be established [9]. MHO is frequently defined as higher than normal BMI (≥25 kg/m^2^) without diabetes mellitus (DM), hypertension (HTN), dyslipidemia, or atherosclerotic cardiovascular disease [10]. However, various investigators employ significantly different criteria to classify MHO. More than 30 definitions of metabolic health were used in various studies to classify MHO, some even including cardiometabolic diseases, and different parameters as a cut-off, including for BMI [11,12,13]. Our study included all patients with no HTN, DM, or dyslipidemia (metabolically healthy) and BMI ≥ 25 in the metabolically healthy obesity (MHO) group and those with BMI < 25 in the metabolically healthy non-obese (MHnO) group as some patients with increased muscle mass can still have increased BMI. 

Conflicting findings have been reported in some studies among MHO and MHNW (metabolically healthy normal weight) individuals with similar risks of CVD in both groups, while others report the opposite. However, systematic reviews and meta-analyses of the accumulating evidence have concluded that individuals with MHO are at increased risk of CVD mortality compared to individuals with MHNW [14,15,16]. Additionally, there is conflicting evidence linking MHO with certain types of cancer, such as colorectal cancer, thyroid cancer in men, endometrial cancer, renal cancer, and post-menopausal breast cancer [17,18,19]. In a meta-analysis by Zheng et al., lower cancer incidence (OR = 0.71; 95% CI: 0.61–0.84) was reported in the MHO phenotype compared to metabolically unhealthy obesity, which is obesity with one of the risk factors (T2DM, HTN, or dyslipidemia) [20]. While MHO individuals may have a lower risk of developing cardiovascular diseases, cancer, and type 2 diabetes mellitus compared to those with metabolically unhealthy obesity, their overall risk is still higher than that of normal-weight individuals [14,21,22,23]. Given the conflicting evidence regarding the relationship between MHO and CVD outcomes, as well as evidence linking MHO to cancers and a limited understanding of its impact on CVD in cancer patients, our present study aims to actively investigate the association and impact of MHO on cardiovascular events in cancer patients. 

## 2. Methods

### 2.1. Data Source

We used data from the National Inpatient Sample (NIS) from 2016 to 2020, the largest all-payer inpatient database available to the public. The Healthcare Cost and Utilization Project (HCUP) created this data source to generate regional and national estimates of inpatient utilization, access, cost, quality, and outcomes in the United States using the International Classification of Disease (ICD-10/relevant codes). The information was extracted using the International Classification of Diseases, Tenth Revision, Clinical Modification (ICD-10-CM) diagnostic codes. Since the NIS dataset is de-identified, it did not require Institutional Review Board (IRB) approval. 

### 2.2. Study Population (Figure 1)

Adult hospitalized cancer patients (age ≥ 18 years) without hypertension, diabetes mellitus, or hyperlipidemia were identified (metabolically healthy individuals). Later, based on the presence or absence of obesity/overweight, they were divided into two groups: metabolically healthy obesity (MHO), those with obesity/overweight (BMI ≥ 25), and metabolically healthy non-obesity (MHnO), those without obesity/overweight (BMI < 25). We used the Elixhauser comorbidity software [24] to compare the prevalence of comorbidities between the two cohorts. Elixhauser comorbidity software uses ICD-10-CM codes in the diagnosis fields and generates the comorbidities as binary variables. We identified patients with acute myocardial infarction (AMI) using ICD-10-CM codes I21.x to I22.x, cardiac arrest (CA) with ICD-10-CM code I46.x, and acute ischemic stroke (AIS) using specific diagnosis code I63.x. For our study, we only looked at the CVD risk factors in defining MHO, as individuals can still have myocardial infarction or stroke despite having no risk factors, so we preferred using a diagnosed CVD risk to define metabolic health rather than a history of MI/stroke for unknown reasons.

**Figure 1:**
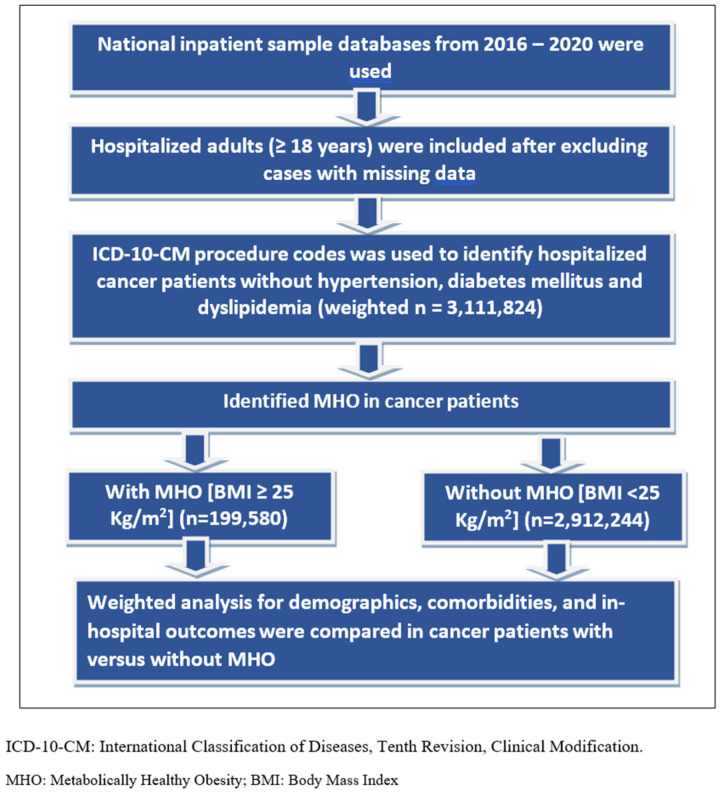
Patient selection and study design.

### 2.3. Study Outcomes

We assessed and compared baseline demographic characteristics (age, sex, race), median household income, payer type, and associated comorbidities (hypertension, diabetes mellitus, hyperlipidemia, obesity, smoking, and alcohol abuse) in both groups (MHO and MHnO). Primary outcomes of interest, mainly in-hospital MACCEs; a composite event that included AMI, CA, AIS, and all-cause mortality (ACM); and individual outcomes were compared between the two cohorts in our study; outcomes were later trended from 2016 to 2020. Secondary outcomes like disposition of patients, length of stay in the hospital (days), and cost of hospital stay (USD) were also compared between the two groups.

### 2.4. Statistical Analyses

We performed the Pearson Chi-square test (categorical data) to compare the baseline characteristics between the two groups (with and without MHO). We used odds ratios, *p*-values, and 95% confidence intervals (CIs) to determine the statistical significance. Outcomes and predictors were adjusted for age, sex, race, median household income, payer type, and outcomes observed in the study population. IBM Statistical Package for Social Sciences (SPSS) v25.0 was used to perform the analyses using complex sample modules, accounting for strata/cluster design. Baseline characteristics between MHO and MHnO cohorts were compared using Pearson’s Chi-square test for categorical variables and Mann–Whitney U test (non-normal distribution) for continuous variables. To reduce the confounding effect, we performed comprehensive multivariable logistic regression analyses, adjusting for sociodemographic characteristics such as sex, race, payer type, median household income, and pre-existing comorbidities like acquired immune deficiency. The trends were assessed using linear-by-linear association tests. We considered a two-tailed-sided *p*-value less than 0.05 as a statistically significant difference. 

## 3. Results

### 3.1. Baseline Characteristics (Table 1)

We identified 3,111,824 cancer-related hospitalizations between 2016 and 2020 that did not involve comorbid conditions like hypertension, diabetes mellitus, and hyperlipidemia. The MHO cohort had 199,580 patients (6.4%), whereas the MHnO cohort had 2,912,244 patients (93.6%). The MHO cohort, with a median age of 57 and a higher proportion of females (64.2% vs. 50.4%; *p* < 0.001), exhibited higher prevalence among Blacks (13.3% vs. 11.0%), Hispanics (12.1% vs. 10.3%), and Native Americans (0.6% vs. 0.5%), but slightly lower prevalence in Whites (72.8% vs. 74.3%) and Asian/Pacific Islanders (1.2% vs. 3.9%) compared to the MHnO cohort, with statistical significance (*p* < 0.001).

**Table 1:** Baseline demographics of hospitalized cancer patients with and without metabolically healthy obesity.

Metabolically Healthy Obesity				*p*-Value
		NO	YES	
Median age in years at admission [IQR range: 25–75]		61 [50–71]	57 [46–66]	<0.001
Sex	Male	1,444,473 [49.6%]	71,449 [35.8%]	<0.001
	Female	1,467,770 [50.4%]	128,130 [64.2%]	
Race	White	2,163,797 [74.3%]	145,294 [72.8%]	<0.001
	Black	320,347 [11.0%]	26,544 [13.3%]	
	Hispanic	299,961 [10.3%]	24,149 [12.1%]	
	Asian or Pacific Islander	113,577 [3.9%]	2395 [1.2%]	
	Native American	14,561 [0.5%]	1197 [0.6%]	
Median household income national quartile for patient ZIP Code	0–25	736,797 [25.3%]	54,685 [27.4%]	<0.001
	26–50	736,797 [25.3%]	53,886 [27.0%]	
	51–75	728,061 [25.0%]	51,092 [25.6%]	
	76–100	710,587 [24.4%]	39,916 [20.0%]	
Hospital bed size	Small	489,256 [16.8%]	33,130 [16.6%]	0.003
	Medium	725,148 [24.9%]	49,296 [24.7%]	
	Large	1,700,750 [58.4%]	117,353 [58.8%]	
Hospital location and teaching status	Rural	168,910 [6.8%]	10,777 [5.4%]	<0.001
	Urban non-teaching	468,871 [16.1%]	29,937 [15.0%]	
	Urban teaching	2,245,340 [77.1%]	158,666 [79.5%]	
Hospital region	Northeast	617,395 [21.2%]	36,922 [18.5%]	<0.001
	Midwest	608,659 [20.9%]	50,693 [25.4%]	
	South	1,057,144 [36.3%]	70,850 [35.3%]	
	West	631,956 [21.7%]	41,712 [20.9%]	
Smoker		774,656 [26.6%]	50,893 [25.5%]	<0.001
Peripheral vascular disease		104,841 [3.6%]	7384 [3.7%]	0.039
Prior MI		43,683 [1.5%]	2994 [1.5%]	0.513
Prior TIA or stroke		66,981 [2.3%]	3592 [1.8%]	<0.001
Hypothyroidism		285,399 [9.8%]	23,949 [12.0%]	<0.001
CHF		189,295 [6.5%]	199,559 [9.8%]	<0.001
Valvular heart disease		17,473 [0.6%]	998 [0.5%]	<0.001
Chronic pulmonary disease		518,379 [17.8%]	39,916 [20.0%]	<0.001
Depression		285,399 [9.8%]	28,141 [14.1%]	<0.001
Prior cancer		486,344 [16.7%]	30,735 [15.4%]	<0.001
Prior chemo		366,942 [12.6%]	25,546 [12.8%]	0.001
Prior RT		279,575 [9.6%]	17,962 [9.0%]	<0.001
**In-Hospital Outcomes:**				
MACCEs [ACM/AMI/CA/AIS]		276,663 [9.5%]	15,767 [7.9%]	<0.001
All-cause mortality		218,418 [7.5%]	12,174 [6.1%]	<0.001
AMI		46,596 [1.6%]	2994 [1.5%]	<0.001
Cardiac arrest		2912 [0.1%]	199 [0.1%]	0.836
AIS		32,034 [1.1%]	1397 [0.7%]	<0.001
Disposition of patient	Routine	1,590,085 [54.6%]	11,1964 [56.1%]	<0.001
	Short-term hospital	72,806 [2.5%]	4790 [2.4%]	
	SNF, ICF	387,328 [13.3%]	26,943 [13.5%]	
	Home healthcare	614,483 [21.1%]	42,510 [21.3%]	
Length of stay (days), median		4	5	<0.001
Cost (USD), median [IQR]		46,658.99	57,085.82	<0.001

IQR—interquartile range, HTN—hypertension, DM—diabetes mellitus, HLD—hyperlipidemia, MI—myocardial infarction, TIA—transient ischemic attack, CHF—Congestive Heart Failure, Prior RT—prior radiotherapy, MACCEs—Major Adverse Cardiovascular and Cerebrovascular Events, AMI—acute myocardial infarction, CA—cardiac arrest, AIS—acute ischemic stroke, SNF—Skilled Nursing Facility, ICF—intermediate care facility.

Comorbidities like peripheral vascular disease (3.7% vs. 3.6%; *p* = 0.039), hypothyroidism (12.0% vs. 9.8%; *p* < 0.001), chronic heart failure (9.8% vs. 6.5%; *p* < 0.001), chronic pulmonary disease (20.0% vs. 17.8%; *p* < 0.001), depression (14.1% vs. 9.8%, *p* < 0.001), and prior chemotherapy (12.8% vs. 12.6%; *p* = 0.001) were more prevalent in the MHO cohort. Both cohorts had a similar prevalence of prior MI (1.5% vs. 1.5%; *p* = 0.513), whereas comorbidities like smoking (25.5% vs. 26.6%; *p* < 0.001), prior transient ischemic stroke (TIA) or stroke (1.8% vs. 2.3%; *p* < 0.001), valvular heart disease (0.5% vs. 0.6%; *p* < 0.001), prior cancer (15.4% vs. 16.7%; *p* < 0.001), and prior radiotherapy (9.0% vs. 9.6%; *p* < 0.001) were less prevalent in the MHO cohort compared to the MHnO cohort.

### 3.2. In-Hospital Outcomes in Cancer Patients with MHO vs. without MHO 

In-hospital MACCEs (7.9% vs. 9.5%; *p* < 0.001), all-cause mortality (6.1% vs. 7.5%; *p* < 0.001), AMI (1.5% vs. 1.6%; *p* < 0.001), cardiac arrest (0.1% vs. 0.1%; *p* = 0.836), and AIS (0.7% vs. 1.1%; *p* < 0.001) were less common in the MHO group compared with the MHnO group. 

### 3.3. Trends in Outcomes from 2016 to 2020 (Table 2)

From 2016 to 2020, among hospitalized cancer patients, there was an increase in the incidence of in-hospital MACCEs [MHO cohort (7.9% vs. 7.9% vs. 7.7% vs. 7.1% vs. 8.9%; Ptrend = 0.002) and MHnO cohort (9.0% vs. 9.2% vs. 9.4% vs. 9.6% vs. 10.5%; Ptrend < 0.001)], all-cause mortality [MHO cohort (6.3% vs. 6.4% vs. 5.9% vs. 5.1% vs. 6.6%; Ptrend = 0.087) and MHnO cohort (7.3% vs. 7.3% vs. 7.3% vs. 7.5% vs. 8.0%; Ptrend < 0.001)], AMI [MHO cohort (1.2% vs. 1.3% vs. 1.4% vs. 1.5% vs. 1.9%; Ptrend < 0.001) and MHnO cohort (1.4% vs. 1.4% vs. 1.7% vs. 1.7% vs. 2.1%; Ptrend < 0.001)], and AIS [MHO cohort (0.6% vs. 0.6% vs. 0.6% vs. 0.9% vs. 0.9%; Ptrend < 0.001) and MHnO cohort (1.0% vs. 1.0% vs. 1.2% vs. 1.2% vs. 1.3%; Ptrend < 0.001)] in both the MHO and MHnO cohorts. However, the incidence of cardiac arrest in the MHO cohort showed a decreasing trend (0.2% vs. 0.25 vs. 0.1% vs. 0.2% vs. 0.1%; Ptrend = 0.013) in contrast to the MHnO cohort, which showed a slightly increasing trend (0.1% vs. 0.1% vs. 0.1% vs. 0.2% vs. 0.25; Ptrend < 0.001) from 2016 to 2020.

**Table 2:** Trends of outcomes from 2016 to 2020 in hospitalized cancer patients with and without metabolically healthy obesity.

Trends of Outcomes		2016	2017	2018	2019	2020	P-Trend
MACCEs	MHO−	9.0%	9.2%	9.4%	9.6%	10.5%	<0.001
	MHO+	7.9%	7.9%	7.7%	7.1%	8.9%	0.002
All-cause Mortality	MHO−	7.3%	7.3%	7.3%	7.5%	8.0%	<0.001
	MHO+	6.3%	6.4%	5.9%	5.1%	6.6%	0.087
AMI	MHO−	1.4%	1.4%	1.7%	1.7%	2.1%	<0.001
	MHO+	1.2%	1.3%	1.4%	1.5%	1.9%	<0.001
Cardiac Arrest	MHO−	0.1%	0.1%	0.1%	0.2%	0.2%	<0.001
	MHO+	0.2%	0.2%	0.1%	0.2%	0.1%	0.013
AIS	MHO−	1%	1%	1.2%	1.2%	1.3%	<0.001
	MHO+	0.6%	0.6%	0.6%	0.9%	0.9%	<0.001

MACCEs—Major Adverse Cardiovascular and Cerebrovascular Events, AMI—acute myocardial infarction, MHO− = without metabolically healthy obesity, MHO+ = with metabolically healthy obesity, AIS—acute ischemic stroke.

### 3.4. Adjusted Odds of Outcomes in Cancer Patients with MHO versus MHnO (Table 3)

In the multivariable logistic regression, adjustments were made for various factors and covariates, including age at admission, sex, race, median household income, drug abuse, chronic pulmonary disease, peripheral vascular disease, smoking status, prior myocardial infarction (MI), prior transient ischemic attack (TIA) or stroke, prior cancer diagnosis, valvular heart disease, anxiety disorders, depression, and prior venous thromboembolism (VTE). After adjusting for these variables, a statistically significant relationship was identified between MACCEs and MHO status in cancer patients.

**Table 3:** Odds of MACCEs in Cancer Patients with vs. without Metabolically Healthy Obesity.

	Odds Ratio	95% Confidence Interval		*p*-Value
		Lower	Upper	
MACCEs	0.93	0.90	0.97	<0.001
All-cause mortality	0.91	0.87	0.94	<0.001
AMI	1.08	1.01	1.16	<0.001
Cardiac Arrest	1.26	1.01	1.57	0.045
AIS	0.76	0.69	0.84	<0.001

Factors and covariates adjusted in multivariable logistic regression included age at admission, sex, race, median household income for the patient’s ZIP Code, drug abuse, chronic pulmonary disease, peripheral vascular disease, smoking status, prior myocardial infarction (MI), prior transient ischemic attack or stroke, prior cancer diagnosis, valvular heart disease, anxiety disorders, depression, and prior venous thromboembolism (VTE). MACCEs—Major Adverse Cardiovascular and Cerebrovascular Events, AMI—acute myocardial infarction, AIS—acute ischemic stroke.

**Figure 2:**
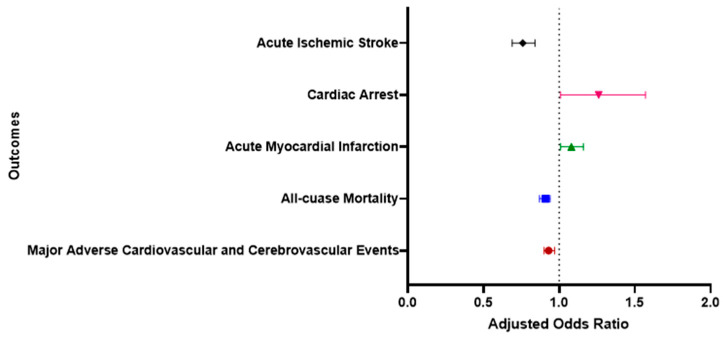
Multivariable analysis for cardiovascular and cerebrovascular outcomes in metabolically healthy obese hospitalized cancer patients.

Upon adjusting for the above variables, there were lower odds of in-hospital MACCEs [adjusted odds ratio (AOR) = 0.93, 95% CI (0.90–0.97), *p* < 0.001], all-cause mortality [AOR = 0.91, 95% CI (0.87–0.94); *p* < 0.001], and AIS [AOR = 0.76, 95% CI (0.69–0.84); *p* < 0.001] in the MHO group. Conversely, the odds for AMI [AOR = 1.08, 95% CI (1.01–1.16); *p* < 0.001] and cardiac arrest [AOR = 1.26, 95% CI (1.01–1.57); *p* = 0.045] were higher in the MHO group (Figure 2). 

## 4. Discussion

In our analysis, among 3,111,824 cancer admissions without HTN, DM, or hyperlipidemia from 2016 to 2020, 6.4% had MHO, of which 35.8% were male. The MHO cohort had a low prevalence (7.9% vs. 9.5%) of MACCEs and other individual outcomes compared to the MHnO cohort. Upon adjusting the variables, the MHO cohort also had decreased odds of in-hospital MACCEs compared to the MHnO cohort. Vague first introduced the MHO concept in the 1950s, and since then, investigating the impact of MHO on cardiovascular diseases has been an ongoing pursuit. Individuals with MHO are defined as obese/overweight individuals with higher than normal BMI (≥25 kg/m^2^) who do not have diabetes mellitus (DM), hypertension (HTN), dyslipidemia, or atherosclerotic cardiovascular disease [10]. However, various investigators employ significantly different criteria to classify MHO. More than 30 definitions of metabolic health were used in multiple studies to classify MHO, some even including cardiometabolic diseases, and different parameters as a cut-off, including for BMI [11,12,13]. Our study included all patients with no HTN, T2DM, or dyslipidemia (metabolically healthy). After that, based on their BMI, the patients were further divided. Patients with BMI ≥ 25 were included in the metabolically healthy obesity (MHO) group, and those with BMI < 25 were included in the metabolically healthy non-obesity (MHnO) group. For our study, we only examined the CVD risk factors when defining MHO. Despite lacking any risk factors, individuals can still experience MI or stroke. Thus, we prefer using diagnosed CVD risk to define metabolic health rather than relying on a history of MI/stroke for unclear reasons. 

The prevalence of the MHO phenotype differs across populations, ranging from 1.1% to 28.5%. This variability could stem from varying definitions of metabolic abnormalities used in studies or other methodological factors, such as small sample sizes or focusing solely on specific subgroups [25]. In a study by Wang et al., among US adults, the age-standardized prevalence of MHO rose from 3.2% (95% CI: 2.6–3.8%) during 1999–2002 to 6.6% (95% CI: 5.3–7.9%) in 2015-2018 [26]. Similarly, in our analysis, among cancer patients, 6.4% had MHO from 2016 to 2020. The differences in the prevalence of MHO in Wang et al. and our analysis compared to other studies can be attributed to the prevalence of obesity in different age groups and the specific subgroups. In recent studies illuminating the growing prevalence of MHO in individuals with cancer, there were questions about the underlying connections between MHO, cancer risk, and cardiovascular outcomes [27]. Obesity intensifies cardiovascular risk through traditional mechanisms, such as dyslipidemia, HTN, glucose dysmetabolism, and other atypical mechanisms [28,29]. The release of inflammatory mediators in obese patients increases oxidative stress and causes endothelial dysfunction [28,29]. A meta-analysis by Aune et al. illustrated a 16% increase in relative risk of cardiovascular death for every 5-unit increment in BMI and a startling 82% surge with each 0.1-unit rise in waist-to-hip ratio [28]. 

The variation in MHO prevalence among different sexes observed in our study can be attributed to the type of cancer prevalence among these patients. Cancers of breast, uterus and testis are based on hormonal profile. Moreover, a few other malignancies are more common in specific genders. A study by Kwon indicated that the incidence of thyroid cancer is twice as high in rate (per 1000 person-years) in females compared to males with MHO [30]. Conversely, bladder cancer is approximately four times more common in men than in women [31]. Contrary to this, in one of their studies, Lin et al. revealed that age and gender do not significantly affect cancer risk among individuals with MHO [18]. Further studies are required to compare cancer incidence rates between males and females and their association with MHO. 

In a study by Fowler et al., which included patients of four different types of cancer (lung, colon, rectum, and Hodgkin’s lymphoma), comorbid conditions like CVD, peripheral vascular disease (PVD), CHF, previous malignancy, and DM were over twice (67%) as prevalent in lung cancer patients when compared to patients with Hodgkin’s lymphoma (30%) [32]. This prevalence consistently increased with age [32]. In female patients among four cancer groups, there were increased adjusted odds of having dementia (29%) and previous malignancy (34%). Notably, females had reduced adjusted odds of having diabetes [OR 0.62; 95%CI: 0.50–0.77)], CVD, and HF when compared to males [32]. Surprisingly, in our study, the MHO cohort, despite its female predominance, had a higher prevalence of PVD, hypothyroidism, CHF, CPD, depression, and prior chemotherapy (64.2%).

Cancer is the second leading cause of death in the US [33]. As per the American Cancer Society, there were around 1.9 million newly diagnosed cases of cancer and 0.6 million cancer-related deaths in 2023 alone [34]. Studies suggest that those with MHO may have a distinct yet favorable inflammatory profile compared to those who are metabolically unhealthy and obese [35]. Despite their obesity, patients with MHO showed no simultaneous presence of metabolic diseases and had a significantly lower cardiovascular risk profile. Previous studies have documented an obesity paradox in cardiovascular outcomes, indicating a decrease in MACCEs among MHO patients [36,37]. Increased lean mass (LM) in obese individuals plays a crucial role. It has been linked to improved long-term outcomes in HF and coronary heart disease patients by boosting cardiorespiratory fitness (CRF) [38]. 

In our study, the odds of in-hospital MACCEs, AIS, and all-cause mortality were lower in the MHO cohort than in the MHnO cohort. Similar findings with decreased cardiovascular mortality (HR: 0.99, 95% CI: 0.93–1.04) were reported in a study conducted in France with a 5-year follow-up. Our study’s lower odds of outcomes can be attributed to the lower proportion of men in the MHO cohort and the obesity paradox. The possible reasons for the obesity paradox in CVDs are because obese patients are often younger with unmeasured confounders, good nutritional reserves, and some patients have increased muscle mass with elevated BMI and fitness levels [39]. BMI alone is not the sole predictor of CVD outcomes, as the patient might have elevated BMI even with increased muscle mass and can be fit, decreasing the risk for CVDs. More appropriate parameters like waist–hip ratio, skeletal weight, muscle mass, and visceral fat are more suitable for predicting these outcomes. 

Moreover, excess adiposity may also offer protection in patients with established CHD. Individuals with higher amounts of LM and concurrent increase in fat mass (FM) tend to have a more favorable prognosis compared to those with high LM and low FM [38]. This increased adiposity appears to be particularly beneficial in individuals with low systemic inflammation, defined as hsCRP < 3 mg/L. Akyea et al. conducted a prospective population-based study following patients with incident cardiovascular disease (CVD) over a median of 13 years. Overweight individuals with no risk factors (RFs) [HR: 0.76 (95% CI: 0.70–0.84)] and obese individuals with no RFs [HR: 0.85 (95% CI: 0.76–0.96)] exhibited a decrease in CVD mortality risk compared to those with a normal BMI and no RFs [40]. Conversely, the likelihood of subsequent non-fatal coronary heart disease (CHD) events and the occurrence of incident heart failure (HF) increased concomitantly with rising BMI and additional metabolic risk factors within each BMI category [40]. 

A sizable observational study exploring the association of MHO with cardiovascular disease and mortality risk suggested that MHO individuals were not at increased risk of CVD or all-cause mortality compared to MHnO individuals. This result persisted even when considering a lean reference group without any metabolic risk factors [41]. The possible reasons for the obesity paradox in CVDs in the MHO cohort can be unmeasured confounders, early presentation of CVD in patients with MHO, and better nutritional reserves, which can be utilized in times of stress [39]. Another theory is that leptin and adiponectin in obese patients have anti-inflammatory properties and can reduce infarct size [42]. However, it is essential to note that BMI alone should not be considered when estimating cardiovascular risk. It can be misleading as BMI does not discriminate between lean and fat mass [43]. In addition, other parameters like waist circumference and waist-to-hip ratio, which can discriminate lean and fat mass more appropriately, should be considered [44]. Even though MACCE prevalence was low in the MHO cohort in our analysis, the median length of hospital stay (five days vs. four days) and cost of stay in the hospital (USD 57,085.82 vs. USD 46,658.99) were high in these patients in our study. 

However, in our study, the odds of AMI were higher in the MHO cohort than in the MHnO cohort. Similar to our study, a meta-analysis and a prospective cohort study also reported an increased risk of CVD in MHO patients [14,21]. However, some studies had not differentiated between individual CVD events and all-cause mortality. Also, these studies were conducted on the general population. Our study included hospitalized cancer patients; no studies have been published on hospitalized cancer patients with MHO. The pathophysiologic mechanisms by which obesity can independently cause atherosclerotic deposition are thought to be primarily due to oxidative stress and a proinflammatory state due to adipocytokines released from adipose tissue [39]. 

Also, MHO is a chronic, recurring condition with a progressive nature, similar to obesity. Individuals with obesity who are on long-term treatment programs fluctuate in weight, transitioning between metabolically unhealthy obesity (MUO) and MHO. Various studies have shown the results of transitioning between MHO and MUO. In a meta-analysis by Lin et al., of 5900 patients who were followed for 3–10 years, at least half developed one metabolic abnormality [45]. In another prospective Pizarra study, nearly 30% of individuals diagnosed with MHO at baseline transitioned to MUO in a 6-year follow-up [46]. In the North West Adelaide Health Study, 16% converted from MUO to MHO in up to 10-year recall visits [47]. 

Studies focusing on the “Fat and fit” phenotype and management focused on chronic inflammation, which is involved in the development of cardiovascular complications, will help stratify patients. More prospective studies and randomized clinical trials focusing on lifestyle interventions in MHO patients with cancer or more granular analysis involving the types of cancer, obesity class in MHO patients, and the severity of MACCEs are needed to understand the impact of MHO in cancer patients on cardiovascular outcomes.

## 5. Strengths and Limitations

One of the strengths of our study is that it is based on a nationwide database comprising a substantial sample size, including patients from a diverse range of demographic backgrounds and geographic locations. As a result, the findings of this study can be applied to patients in different healthcare settings. It also helps establish clear inferences and allows for the generalizability of the findings. 

However, our study has certain limitations, and the results need to be interpreted considering these limitations. Since NIS data are from an inpatient-only database, certain in-built flaws are possible, such as information bias, selection bias, and confound bias. The data in our study do not include the baseline lifestyle factors of the individuals, the severity of in-hospital MACCEs, the type of cancer, or other characteristics that can modify the outcomes. This study is a retrospective study that could not establish causality and should be considered hypothesis-generating. 

We used BMI solely to differentiate between MHO and MHnO groups, and we included both obesity and overweight patients in the MHO group (BMI ≥ 25) in this study. Neither waist-to-hip ratio nor waist circumference were available to define obesity/overweight. Also, data are not available regarding the transitions between MHO, MUO, and MHNW among patients during hospitalization, which can affect outcomes and can be the focus of future studies. 

## 6. Conclusions

Hospitalized cancer patients with MHO exhibited a lower prevalence of MACCEs compared to those with MHnO. Upon adjusting for covariates, an obesity paradox emerged in the MHO cohort, revealing decreased odds in MACCEs, all-cause mortality, and AIS compared to the MHnO cohort. To validate these findings, additional prospective studies and randomized clinical trials are imperative, particularly in stratifying MHO across various cancer types and their corresponding risks of MACCEs.

## Data Availability

This published article includes all data generated or analyzed during this study.
